# Fuchs' Endothelial Corneal Dystrophy in Patients With Myotonic Dystrophy, Type 1

**DOI:** 10.1167/iovs.17-23160

**Published:** 2018-06

**Authors:** Nelson S. Winkler, Margherita Milone, Jennifer M. Martinez-Thompson, Harish Raja, Ross A. Aleff, Sanjay V. Patel, Michael P. Fautsch, Eric D. Wieben, Keith H. Baratz

**Affiliations:** 1 Department of Ophthalmology, Mayo Clinic, Rochester, Minnesota, United States; 2 Department of Neurology, Mayo Clinic, Rochester, Minnesota, United States; 3 Mount Sinai School of Medicine, New York, New York, United States; 4 Department of Biochemistry and Molecular Biology, Mayo Clinic, Rochester, Minnesota, United States

**Keywords:** cornea, corneal dystrophies, corneal genetics, Fuchs' endothelial corneal dystrophy, myotonic dystrophy

## Abstract

**Purpose:**

RNA toxicity from CTG trinucleotide repeat (TNR) expansion within noncoding DNA of the transcription factor 4 (*TCF4*) and DM1 protein kinase (*DMPK*) genes has been described in Fuchs' endothelial corneal dystrophy (FECD) and myotonic dystrophy, type 1 (DM1), respectively. We prospectively evaluated DM1 patients and their families for phenotypic FECD and report the analysis of CTG expansion in the *TCF4* gene and *DMPK* expression in corneal endothelium.

**Methods:**

FECD grade was evaluated by slit lamp biomicroscopy in 26 participants from 14 families with DM1. CTG TNR length in *TCF4* and *DMPK* was determined by a combination of Gene Scan and Southern blotting of peripheral blood leukocyte DNA.

**Results:**

FECD grade was 2 or higher in 5 (36%) of 14 probands, significantly greater than the general population (5%) (*P* < 0.001). FECD segregated with DM1; six of eight members of the largest family had both FECD and DM1, while the other two family members had neither disease. All DNA samples from 24 subjects, including four FECD-affected probands, were bi-allelic for nonexpanded TNR length in *TCF4* (<40 repeats). Considering a 75% prevalence of *TCF4* TNR expansion in FECD, the probability of four FECD probands lacking TNR expansion was 0.4%. Neither severity of DM1 nor *DMPK* TNR length predicted the presence of FECD in DM1 patients.

**Conclusions:**

FECD was common in DM1 families, and the diseases cosegregated. *TCF4* TNR expansion was lacking in DM1 families. These findings support a hypothesis that *DMPK* TNR expansion contributes to clinical FECD.

Fuchs' endothelial corneal dystrophy (FECD) is strongly associated with variation in the transcription factor 4 (*TCF4*) gene in the majority of cases in the United States^1–7^ and Australia^8^ and a minority of cases in Asia.^9–12^ Our current understanding of the disease indicates that RNA toxicity due to an intronic (noncoding) CTG trinucleotide repeat (TNR) expansion in *TCF4* may underlie the pathogenesis of FECD.^13,14^ This disease mechanism is shared with the neuromuscular degeneration, myotonic dystrophy type 1 (DM1), which also harbors the same CTG TNR expansion within the 3′ untranslated region in the DM1 protein kinase (*DMPK*) gene.^15^ Intranuclear accumulation of premessenger RNA transcribed from the CTG repeats leads to the sequestration of critical RNA-splicing factors, such as proteins of the muscleblind family, which results in widespread alterations in mRNA splicing and altered protein isoform populations.^13,16–19^

Because the mechanism of CTG repeat–induced RNA toxicity may be at least partially independent of the chromosomal location of the TNR expansion within the genome, there exists the possibility that FECD and DM1 may share phenotypic features. There is no established association between FECD due to *TCF4* repeat expansion and neuromuscular signs or symptoms, but prior reports have been mixed regarding corneal findings in patients with DM1. Two initial studies described normal corneal thickness in patients with DM1,^20,21^ and a follow-up study by Rosa and colleagues^22^ found an increased corneal thickness, normal endothelial cell density, and a lower coefficient of variation in a cohort of patients with DM1. Gattey and colleagues^23^ described typical clinical and histologic features of FECD in four patients from three families with established DM1, and Heringer and colleagues^24^ reported two cases of FECD in patients with DM1 from one family, though genetic analysis of *TCF4* and *DMPK* were not presented in either study. In a preliminary study, we confirmed an association between FECD and DM1 (Winkler N, et al. *IOVS* 2017;58:ARVO E-Abstract 3796). Recently Mootha and colleagues^25^ found a high prevalence of phenotypic FECD in 6 of 13 unrelated patients with DM1.

The specific aims of the current study were to prospectively evaluate DM1-affected patients and their families for the presence of phenotypic FECD and to report the analysis of CTG expansion in the *TCF4* gene. We also examined corneal endothelial tissue retrieved at endothelial keratoplasty in a separate study for *DMPK* gene expression.

## Methods

Patients with DM1 and their family members were recruited from the Department of Neurology clinical practice at Mayo Clinic in Rochester, Minnesota. After informed consent, a total of 26 participants from 14 families underwent slit lamp biomicroscopy by ophthalmology investigators (KHB, SVP) in order to identify the presence of corneal guttae, which were graded using the modified Krachmer scale (0, no guttae, through 6, confluent guttae with corneal edema).^26,27^ A Krachmer grade ≥2 was considered to be indicative of FECD. Phlebotomy was performed, and DNA was extracted from leukocytes.

Analysis for repeat expansion in *TCF4* in all participants was performed by using a combination of a short tandem repeat assay of PCR-expanded DNA and Southern blotting of unexpanded genomic DNA from leukocytes as described previously.^1^ Briefly, CTG repeat length for both alleles was quantified by Gene Scan analysis (GeneScan; Applied Biosystems, Foster City, CA, USA). Samples in which only one repeat length was identified were further assayed by Southern blotting to confirm the presence of either a single repeat length or the presence of a larger expansion (>120 CTG repeats) that was too large to be detected by Gene Scan. The threshold for a TNR expansion was established at >40 CTG repeats.

In order to evaluate corneal endothelial tissue for DMPK expression, tissue was obtained at the time of endothelial keratoplasty for Fuchs' dystrophy or postoperative corneal edema from patients enrolled in a separate protocol approved by the Mayo Clinic Institutional Review Board and conducted in accordance with the tenets of the Declaration of Helsinki. RNASeq was used to determine gene expression within the corneal endothelium as previously described.^28^ Briefly, total RNA was isolated and RNA libraries were prepared, with RNA integrity number values of ≥6.0. Libraries were minimally amplified to enrich for fragments and then quantified for sequencing at three samples per lane by using a sequencer (HiSeq4000; Illumina, San Diego, CA, USA). Additional details are available elsewhere.^28^

A diagnosis of DM1 in probands was established by a neurologic evaluation (MM, JMM-T) and genetic analysis of the *DMPK* gene, provided by an external laboratory (Athena Diagnostics, Inc., Marlborough, MA, USA). The presence or absence of DM1 in family members was established based on their clinical and laboratory history.

## Results

Of the 14 probands with DM1 (six male, eight female) examined, five probands (two male, three female) had clinical FECD (36%). This prevalence differs (*P* < 0.01) from the general population, previously reported to be 5% of individuals over the age of 40 in the United States.^29^ The severity of FECD ranged from 2 (mild disease) to 6 (the most severe grade). There was a wide age range (22–68 years), with one patient demonstrating signs of FECD at age 22 (Table and Supplementary Table).

**Table:**
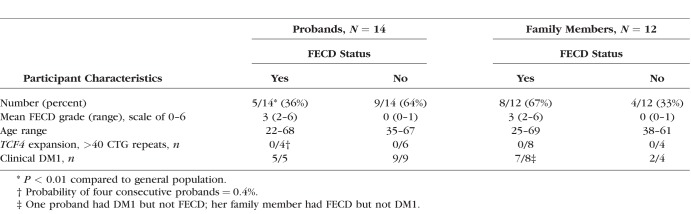
Characteristics of Probands With Myotonic Dystrophy, Type 1 and Their Family Members

Blood samples were obtained from four of the five probands, and all four demonstrated a lack of CTG expansion in *TCF4*. Given the prevalence of a *TCF4* TNR expansion in 75% of all FECD patients in our cohort,^1^ the probability of four consecutive patients without repeat expansion in this gene is 0.4%. By definition, all study probands had TNR expansion in the *DMPK* gene (range, 85–663).

**Figure 1:**
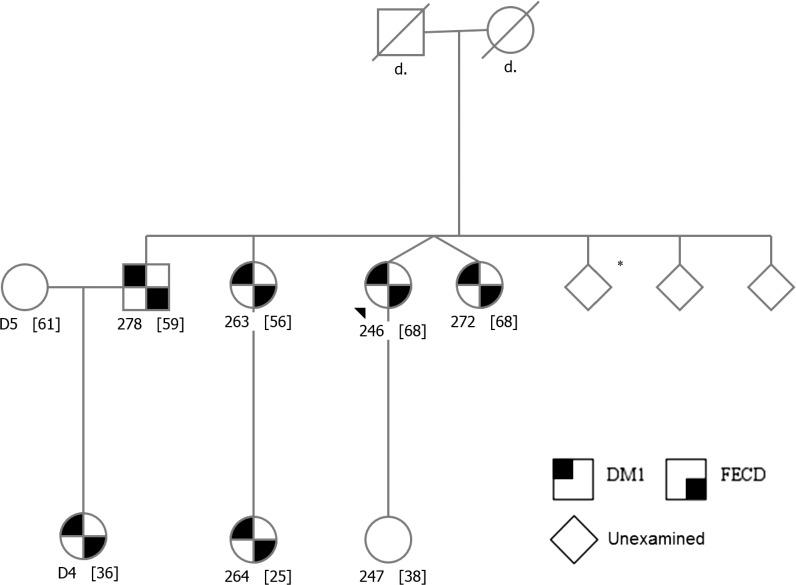
Family pedigree demonstrating cosegregation of DM1 with FECD. All family members with DM1 have FECD, and those without DM1 did not show evidence of FECD. Subject ID [Age]; *DM1 by history only.

In addition to the 14 probands, we examined 12 family members and found cosegregation of DM1 and FECD status, as illustrated in the largest family pedigree in Figure 1 and in Supplementary Figures. Of the eight family members (one male, seven female) who were related to FECD-affected probands, six (one male, five female) had both DM1 and FECD and two (both female) had neither DM1 nor FECD. The youngest family member with FECD was 25 years old. Of the four family members who were related to FECD-unaffected probands, two (one male, one female) had DM1 but not FECD, one female had neither, and one subject (female, 69-years-old) had grade 2 FECD but did not have DM1. No proband or family member had TNR expansions within the *TCF4* gene. There was no correlation between FECD status and any DM1 clinical or genetic characteristics. The mean *DMPK* repeat length of FECD-affected and FECD-unaffected probands was 324 (*n* = 5, SD ± 216) and 490 (*n* = 7, SD ± 254; *P* = 0.265, 2-tailed *t*-test), respectively.

**Figure 2:**
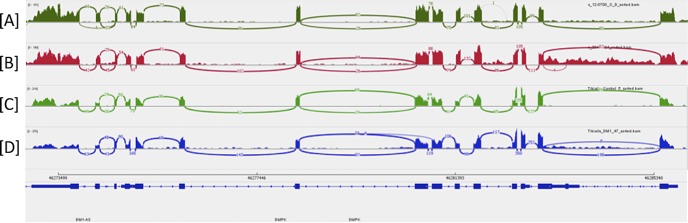
Sashimi plot of RNASeq data confirming gene expression within the DMPK gene. (A) Corneal endothelium from a patient undergoing keratoplasty for pseudophakic bullous keratopathy, (B) corneal endothelium from a patient with TCF4 repeat expansion–associated FECD, (C) normal striated muscle, and (D) DM1 striated muscle. The vertical axis for each plot depicts the number of sequencing reads that map to that location in the genome. Muscle data were downloaded from the GEO DataSet GSE86356 (available in the public domain, https://www.ncbi.nlm.nih.gov/gds/?term=GSE86356%5BAccession%5D). Note that the coordinates for this gene displayed at the bottom of the figure are from UCSC Genome Browser (available in the public domain, https://genome.ucsc.edu) Human Genome GRCh37/hg19.

**Figure 3:**
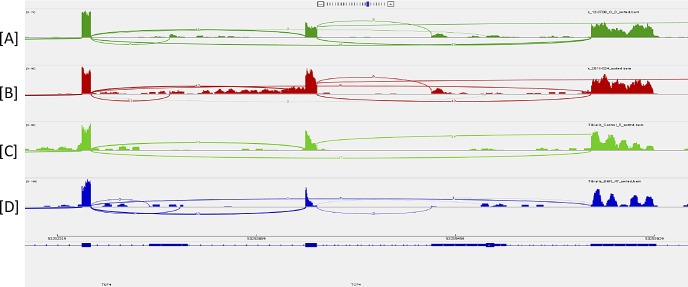
Sashimi plot of RNASeq data confirming gene expression within the TCF4 gene in the same samples as Figure 2, including (A) corneal endothelium from a patient undergoing keratoplasty for postoperative corneal edema, (B) corneal endothelium from a patient with TCF4 repeat expansion–associated FECD, (C) normal striated muscle, and (D) DM1 striated muscle. The vertical axis for each plot depicts the number of sequencing reads that map to that location in the genome. Muscle data were downloaded from the GEO DataSet GSE86356 (available in the public domain, https://www.ncbi.nlm.nih.gov/gds/?term=GSE86356%5BAccession%5D). Note that the coordinates for this gene displayed at the bottom of the figure are from UCSC Genome Browser (available in the public domain, https://genome.ucsc.edu) Human Genome GRCh37/hg19.

RNASeq analysis of RNA from the corneal endothelium of a patient with pseudophakic bullous keratopathy and a patient with TCF4 expansion–associated FECD demonstrated robust expression of the *DMPK* (Fig. 2) and *TCF4* (Fig. 3) genes in this tissue in both samples. Expression of these genes in striated muscle samples was confirmed from online Gene Expression Omnibus (GEO) DataSets (available in the public domain, https://www.ncbi.nlm.nih.gov/gds) and is also shown in Figures 2 and 3.

## Discussion

The prevalence of FECD in this cohort of patients with DM1 (36% of probands) was similar to a previous study (46%)^25^ and higher than that in the general population. Among families that demonstrated FECD, we found that the FECD phenotype consistently cosegregated with DM1; participants who had FECD also had DM1, and those without DM1 did not have FECD. Among families in which the proband did not have FECD, one family member had mild FECD but not DM1. Furthermore, CTG TNR expansion in *TCF4*, which is present in approximately 75% of FECD patients, was not present in any of the study participants. Taken together, the high prevalence of FECD in DM1 patients, the cosegregation of the disease phenotypes, the lack of TCF4 repeat expansion, and the robust expression of the *DMPK* gene in the corneal endothelium (Fig. 2), are compatible with a hypothesis that the *DMPK* gene may cause the FECD phenotype in some but not all patients with DM1. This is further supported by findings by Mootha and colleagues,^25^ who described colocalization of MBNL-1 and CUG RNA within RNA foci, the histologic hallmark of CTG repeat-mediated disease, in the endothelium of an eye bank donor with DM1. Identical foci have been found in the corneal endothelium of FECD patients harboring *TCF4* TNR expansion.^13^ A less likely explanation for our findings is that the cosegregation of DM1 and FECD in the absence of *TCF4* repeat expansion is purely coincidental or that the FECD is induced by a previously unrecognized genetic variant in linkage disequilibrium with the causative *DMPK* expansion. To our knowledge, no other associated genetic variant on chromosome 19q has been described in FECD.

In addition to DM1, other repeat expansion diseases are all neurologic and neuromuscular degenerations, and the molecular mechanisms of these diseases, which includes Huntington disease, spinocerebellar ataxia, and fragile X syndrome, are well defined. Short repeat lengths are not pathologic, but larger repeat lengths are unstable and cause disease through a variety of mechanisms, such as RNA toxicity or by translation into protein. In the *DMPK* gene, expanded CTG repeats result in the accumulation of transcribed CUG pre-mRNA, which binds and sequesters the RNA-splicing proteins, muscleblind 1 and 2, into intranuclear foci. The inactivation of the muscleblind proteins leads to a pattern of widespread RNA mis-splicing, which is ultimately responsible for the systemic disease phenotype of progressive muscle weakness with myotonia, cognitive dysfunction, cardiac conduction defects, and premature cataracts.^16–19^

In spinocerebellar ataxia, it has been shown that patients have a decreased corneal endothelial cell density.^30^ Recently, Campos-Romo and colleagues^31^ demonstrated an inverse relationship between the number of CAG TNRs and endothelial cell density, as well as an association between disease severity and decreased endothelial cell density. Our RNASeq analysis revealed that the spinocerebellar ataxia–associated gene ataxin 7 (*ATXN7*) is expressed in the corneal endothelium, which suggests the possibility of a mechanism of disease similar to that found in FECD and DM1.

It is not surprising that we did not find an association between DM1 clinical or genetic characteristics and the presence or severity of FECD. Although such correlations may exist, we would not expect to find a link between the severity of FECD and a complex, progressive multisystem disease in this small study. As in other inherited disorders, variation of phenotype expression and severity within a pedigree and between pedigrees is typical. Discovering such correlations would require a larger study cohort and multivariate analysis, which is beyond the scale of this study. Additionally, TNRs are unstable, so the repeat length in leukocytes may not correlate to the repeat length in affected tissues.^32^ We are unable to explain why some but not all families with DM1 demonstrate the FECD phenotype. Our assumption is that other unidentified genetic factors play a role in phenotype expression. In our other studies of FECD, we have found several subjects with TCF4 repeat expansions that do not have clinical disease.^1^

In summary, current data strongly support the premise of a shared pathophysiologic mechanism of RNA toxicity between FECD and DM1 and suggests that the pathogenic contribution of CTG repeat expansion may be independent of the location of the expansion within the genome. The feasibility of DMPK repeat expansion as the etiology of the FECD phenotype is corroborated with DMPK expression data in corneal endothelium and the cosegregation of the two phenotypes within pedigrees. Further studies should be directed toward the ongoing examination of corneal endothelium from ex vivo samples or cell culture for additional evidence of RNA toxicity in DM1 and other TNR expansion diseases.

## Supplementary Material

Supplement 1

Supplement 2
