# Supplementary material for: Fuchs' Endothelial Corneal Dystrophy in Patients With Myotonic Dystrophy, Type 1
Source: Invest Ophthalmol Vis Sci. 2018 Jun;59(7):3053–7. doi: 10.1167/iovs.17-23160 (PMC6005624; doi:10.1167/iovs.17-23160)
Supplement: Supplement 1 [file iovs-59-06-65_s01.pdf]

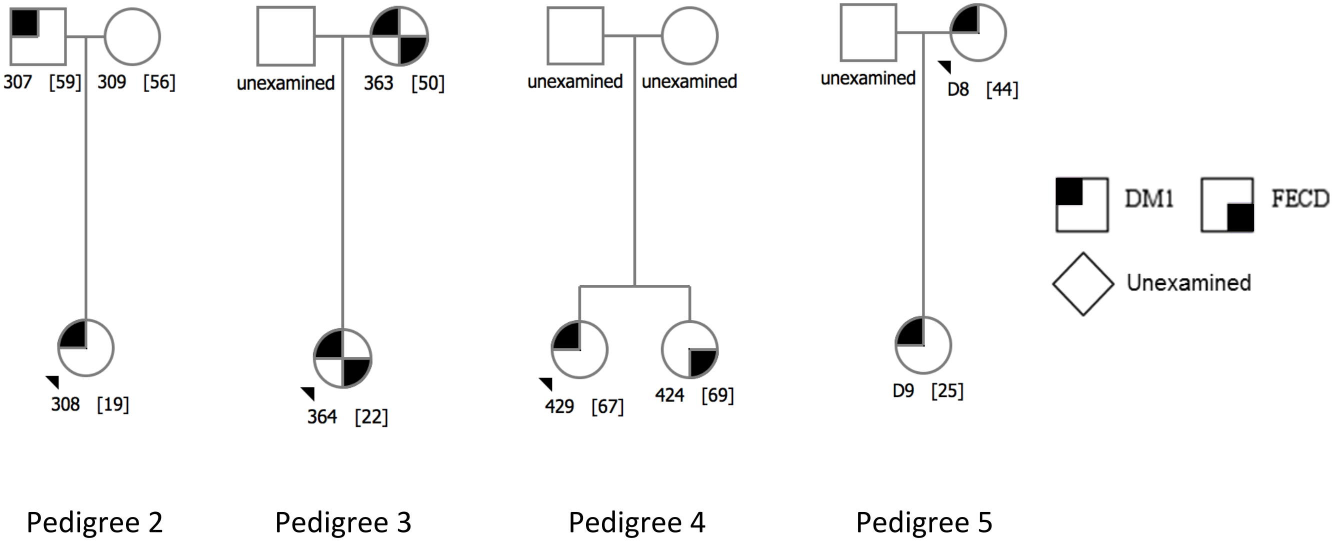

307 [59]
309 [56]
308 [19]
Pedigree 2
unexamined
363 [50]
364 [22]
Pedigree 3
unexamined
unexamined
429 [67]
424 [69]
Pedigree 4
unexamined
D8 [44]
D9 [25]
Pedigree 5
DM1
FECD
Unexamined

**Supplemental Figure.** Family pedigrees of related subjects demonstrating co-segregation of myotonic dystrophy, type 1 (DM1) with Fuchs endothelial corneal dystrophy (FECD). Labels: Subject ID [Age].
