# Supplementary material for: Fuchs' Endothelial Corneal Dystrophy in Patients With Myotonic Dystrophy, Type 1
Source: Invest Ophthalmol Vis Sci. 2018 Jun;59(7):3053–7. doi: 10.1167/iovs.17-23160 (PMC6005624; doi:10.1167/iovs.17-23160)
Supplement: Supplement 2 [file iovs-59-06-65_s02.pdf]

Supplemental Table. Demographic, Microscopy, and Repeat Expansions of DM1 Patients

					Krachmer Grade		TCF4 Alleles			DMPK Alleles		
**Subject**	**Sex**	**Age**	**Proband or Relationship**	**FECD Status**	**OD**	**OS**	**Short**	**Long**	**DM1 Status**	**Short**	**Long**	**Cataract or Lens Implant**
PEDIGREE 1												
246	F	68	proband	Y	6	6	18	27	Y	11	85	Y
272	F	68	identical twin	Y	6	6	17	26	Y		expanded***	Y
278	M	59	brother	Y	unable	2.5	15	25	Y	11	103	Y
247	F	38	daughter	N	0	0	27	29	N		not expanded***	N
263	F	56	sister	Y	4	5	26	26*	Y		expanded***	N
264	F	25	niece	Y	2	2.5	27	27*	Y		expanded***	N
D4	F	36	niece	Y	4	4	19	25	Y	13	783	Y
D5	F	61	sister-in-law	N	1	1	12	19	N		not expanded***	N
PEDIGREE 2												
308	F	19	proband	N	0	0	18	23	Y	15	253	N
307	M	59	father	N	0	0	18	25	Y		expanded***	N
309	F	56	mother	N	0	0	18	23	N		not expanded***	N
PEDIGREE 3												
364	F	22	proband	Y	2	2	12	12*	Y	unknown	450	Y
363	F	50	mother	Y	4	3	12	12*	Y	11	83	Y
PEDEGREE 4												
429	F	67	proband	N	0	0	12	19	Y		expanded***	Y
424	F	69	sister	Y	2	2	12	19	N		unknown	Y
PEDIGREE 5												
D8	F	44	proband	N	0	0	12	12*	Y	11	500	N
D9	F	25	daughter	N	0	0	12	15	Y	11	800	N
UNRELATED SUBJECTS												
D3	M	48	proband	Y	2	2	12	17	Y	13	230	Y
315	F	39	proband	Y	4	4	23	25	Y	11	223	N
D10	M	39	proband	Y	2.5	2.5	**	**	Y	14	633	Y
D1	M	56	proband	N	0	0	19	26	Y	5	377	Y
D2	F	54	proband	N	0	0	12	12	Y	20	376	N
341	F	35	proband	N	0	0	12	23	Y	5	800	Y
389	M	44	proband	N	0	1	**	**	Y	5	183	Y
D6	M	41	proband	N	0	0	15	17	Y	5	790	Y
D7	M	35	proband	N	0	0	12	16	Y	14	650	Y

* Genescan analysis disclosed only one allele length. The absence of an expanded allele was confirmed by Southern Blotting, indicating that both alleles are the same length

** No specimen available

***Data provided via verbal medical history
